# Proportion, Pattern and Need Assessment of Deformities Among Registered Leprosy Affected Individuals in Chamrajanagar District

**DOI:** 10.4103/0970-0218.66885

**Published:** 2010-04

**Authors:** Gautham M Sukumar, NS Shivaraj, M Dayananada

**Affiliations:** Department of Community Medicine, M S Ramaiah Medical College, Bangalore, Karnataka, India

## Introduction

India achieved success in the elimination of leprosy (0.95/10000 population) by December 2005.(1) Elimination is not eradication and leprosy cases will continue to present even after the disease has been declared as eliminated. With epidemiological shift from elimination to post-elimination phase, surveillance systems should be strengthened at all levels to ensure that appropriate information on leprosy patients is collected and managed.(2)

Chamrajanagar district had prevalence rate as high as 89/10,000 population at the time of beginning MDT, following which there was a sustained decline in the prevalence. Shifting the case detection strategy post integration, from active case detection to voluntary reporting, has resulted in decreased opportunities for health system and patient contact. In final stages of the battle against this disease, pertinent epidemiological information regarding changes in disease characteristics and distribution is vital to monitor progress of elimination and formulate interventions. Hence, a study was conducted with the following objectives:

- To assess the proportion and pattern of deformities among registered leprosy affected individuals in Chamrajanagar district; and
- To assess the care needs for disability limitation and rehabilitation among leprosy affected individuals having deformity.

## Materials and Methods

This community-based, cross-sectional study was conducted within the administrative limits of Chamrajanagar district, Karnataka. Permission was obtained from the State and District Leprosy Office and clearance from ethical committee of M S Ramaiah Medical College. A pilot study was conducted in a sample of LAI’s from selected PHCs and a situation analysis was made from the existing records in the District Leprosy Office.

From the records, information and address of all LAI’ registered from 1^st^ April 2005 to 31^st^ December 2006 and utilized/utilizing services from the leprosy treatment units and residing within the district were obtained and included for the study. Each of the available registered case was contacted between January 2007 and May 2007; informed consent was obtained and interviewed/examined using a pretested, semi-structured questionnaire-cum-examination checklist. Individuals not residing within the administrative limits of the district and those not willing to participate were excluded.

Information was collected on parameters like socio-demographics, deformity assessment, grading and self care. Deformity assessment was based on WHO Guidelines for Assessment and Grading for Disability in Leprosy.(3) WHO Grading is as Grades: 0, 1 and 2. Grade 0: no deformity. Grade 1: loss of sensation but no visible deformity. Grade 2: presence of visible deformity. Examination of the patient, type of care needed and assessment of self-care needs was carried out based on standard guidelines adapted from ”Guidelines for implementing a disability prevention programme in the field” by H Srinivasan.(4)

The investigator had received training from the District Technical Support Team (DTST) to assess deformity, and the findings in sample of patients were validated by the DTST staff.

## Results and Discussions

Situation analysis: Records at the District Leprosy Office (DLO) revealed that leprosy had attained elimination levels in 2005-2006 (i.e. PR=0.84/10,000 population) but has witnessed increase in prevalence in 2006-2007 (1.03/10,000 population). Deformity proportion among the new cases was 0.62% in 2005-2006 and 4.94% at 2006-2007 and the number of deformity cases had increased more than eight times since last two years.

### Observations

General: There were 275 registered LAI’s from April 2005 to December 2006. 259 (95%) were available for the study (16 cases were not available due to migration for work, not able to contact, left area permanently). 55.6% of the LAI’s were males, 34.4% were multibacillary cases and nearly 50% were illiterate. 38.2% of the LAI’s were currently under treatment and 61.8% were released from treatment.

### Deformity proportion and pattern

In leprosy circles, the terms deformity and disability are used synonymously and graded as Grade 0, 1 or 2.(3) Table 1 shows that 8.5 % had Grade 1 (loss of sensation) and 11.6% had Grade 2 (visible deformity). Among the Grade 2 deformity the pattern of deformities observed was the following: ulcers in hands 17(56.7%), claw hand 18 (60%), scars/cracks in hands 17(56%), scars/cracks in hands 17(56%) and plantar ulcers 6 (20%) and foot drop 1(3.3%)

**Table 1:** Distribution of study subjects according to WHO grading of disability

WHO Grading of disability*	Frequency (%)
Grade 0	207 (79.9)
Grade 1	22 (8.5)
Grade 2	30 (11.6)
Total	259 (100)

* Grade 0= No deformity, Grade 1= loss of sensation, Grade 2=visible deformity

Literature review shows that Grade II deformity was found in 4% of newly detected cases in 2000 worldwide, 3% in SEAR, 1.8% in India (2004).(5) Our field level assessment showed a higher deformity proportion probably as both existing and release from treatment cases were studied. A study in Gwalior revealed that 35% of the cases had Grade 2 disability.(6)

Grade 1 deformity which is precursor for developing Grade 2 deformities is not currently recorded. Many cases could have developed deformity after being diagnosed or after starting treatment, post-reactions or even after release from treatment. Post-integration, there was a lack of system for training to neither assess deformity nor progressively monitor patients for deformity assessment post-treatment. These are issues that need to be addressed if elimination is to be made meaningful to address quality of life and not only mere completion of treatment.

### Need assessment

Skin, wound and joint care was the predominant type of care needed by the study subjects [Table 2].

**Table 2:** Type of care needs among LAI’s with deformity

Care needs	Frequency (N=52)
Skin care	52 (100)
Wound care	23 (44.23)
Joint care	19 (36.53)
Swelling care	4 (7.6)
Eye care	1 (0.19)
Nerve care	9 (17.3)

*Above table includes individuals with multiple care requirements

There is an unmet training need as less than 30% of the subjects were trained in activities of self-care and even a smaller fraction actually practised it, as evident from Table 3.

**Table 3:** Self-care practices among LAI’s with deformity

Activity	Trained	Practices	Have requisite tools
Soaking, scrubbing smearing oil, and dressing (SSOD) (n=52)	13 (25)	8 (15.3)	10 (19.2)
Use of protective devices (n=52)	28 (53.8)	16 (30.7)	14 (26.9)
Clean and dress wound/ulcer/crack (n=23)	6 (26.1)	2 (8.7)	2 (8.7)
Joint care practices (n=19)	5 (26.3)	3 (15.8)	-

Figures in parenthesis are percentages

## Conclusion

Deformity proportion is increasing in the district and there is a need to monitor LAI’s for deformity (both Grade 1 and Grade 2) during treatment and perhaps even after treatment completion. Voluntary detection as strategy may have delayed detection of deformities. The needs of the LAI’s were identified as training and resources in self-care (skin care, wound care and joint care), provision of necessary protective equipment, orthopedic appliances, physiotherapy, mobility aid and reconstructive surgery.

Recommendations: Post integration era, it is an urgent unmet need to step up targeted, need- based behavioral change communication (BCC) strategies which will influence detection delay, early recognition and management of reactions, promote early reporting and self-care practices in the community.

It is recommended to conceptualize and implement a field-based disability care and prevention program and capacity building of health personnel at all levels-community-based rehabilitation. As an immediate measure, it is recommended to conduct POD sessions for all health care workers and leprosy cases having Grade 1 and 2 disabilities with monthly monitoring of patients. Procurement, distribution and training should be given to use the tools for self-care for the identified LAI’s with support of Disability Prevention and Medical Rehabilitation (DPMR) under NLEPIII.

Government, NGOs and private organizations need to work together in a coordinated fashion in the final battle against leprosy to avoid any complacency in this assumed success in elimination. The natural history of disease is still uncertain and immediate need of the hour is to consolidate the gains achieved.
